# Ethics beyond ethics: the need for virtuous researchers

**DOI:** 10.1186/s12910-018-0281-6

**Published:** 2018-06-15

**Authors:** Mark Daku

**Affiliations:** 1 Department of Political Science, Texas Christian University, TCU Box 297021, Fort Worth, 76129 TX USA; 2 Institute for Health & Social Policy, McGill University, 1130 Pins Ave. W., Montreal, Canada

**Keywords:** Ethics, Virtue ethics, Research ethics boards, South Africa, Drinking and driving

## Abstract

**Background:**

Research ethics boards (REBs) exist for good reason. By setting rules of ethical behaviour, REBs can help mitigate the risk of researchers causing harm to their research participants. However, the current method by which REBs promote ethical behaviour does little more than send researchers into the field with a set of rules to follow. While appropriate for most situations, rule-based approaches are often insufficient, and leave significant gaps where researchers are not provided institutional ethical direction.

**Results:**

Through a discussion of a recent research project about drinking and driving in South Africa, this article demonstrates that if researchers are provided only with a set of rules for ethical behaviour, at least two kinds of problems can emerge: situations where action is required but there is no ethically good option (*zungzwang* ethical dilemmas) and situations where the ethical value of an action can only be assessed after the fact (*contingent* ethical dilemmas). These dilemmas highlight and help to articulate what we already intuit: that a solely rule-based approach to promoting ethical research is not always desirable, possible, effective, or consistent.

**Conclusions:**

In this article, I argue that to better encourage ethical behaviour in research, there is a need to go beyond the rules and regulations articulated by ethics boards, and focus more specifically on creating and nurturing virtuous researchers.

## Background

It was sometime around midnight, and the bar was closing. Some of the more committed drinkers remained inside, the bar owners locking the doors to transform the public bar into a private party—a common trick to get around liquor laws. On the street people milled about, chatting, flirting, slowly making their way home or to whatever was next. As I walked by, one woman was lying on the ground, clearly inebriated and yet not ready to give up on the girl she had been flirting with all night. She tried to push her way to a standing position and asked the girl to come home with her, pointing to her car parked a few feet away.

I had been coming to this bar for several years and had been attentively observing it for the past few weeks as part of a research project on drinking and driving in South Africa. Drunk driving was not rare in this space; in fact, it was considered normal. This was, however, a case of a different magnitude. As she was unable to stand, I was sure this woman would be a tremendous risk to herself and others if she were to attempt to drive home (a 30-min drive to her neighbourhood, give or take).

As a private citizen, I would have been free to attempt to defuse the situation any way I saw fit. I could have simply stolen her keys, or called the police, or called on the bar staff to stop her from driving (or have them call the police). However, I was not in the situation as a private citizen. I was there as a researcher and as such was bound by particular rules I needed to follow to conduct my research, protect my participants and myself, and do so ethically. These rules, however, had been set over 13,000 km away in Canada, by an institution whose representatives had likely never set foot in South Africa or tried to do research in this particular bar.

In its efforts to determine the ethical principles that should guide research, the Belmont report notes that rules “often are inadequate to cover complex situations; at times they come into conflict, and they are frequently difficult to interpret or apply” [1]. The issue that I hope to highlight in this article is that the principles that are embedded in the Belmont report, principles about respect for persons, beneficence, and justice, make their way to the researcher exclusively through the REBs in the form of rules and restrictions. Guidelines for ethical research, particularly medical research, can produce a preponderance of correct procedures and rules, “lists and check-boxes that potentially undermine rather than promote vigorous and critical ethical debate...” ([2], p. 68). Furthermore, debates about research ethics “tend to be dominated by the views of scientists and advocates from high-income settings” ([2], p. 68). In other words, ethical principles become codified as rules for research, however those rules are not necessarily appropriate for any specific research context. While there is a recognition that rules are not enough, in practice, researchers interact with a solely rule-based institutional ethical environment.

Through a discussion of a recent research project in South Africa, I hope to clearly demonstrate and articulate the inadequacy of a solely rule-based approach to ethical research, and I advocate supplementing that approach with a virtue-based approach to preparing researchers for their work. Herein, I argue that while necessary and sufficient in many cases, approaches the rely solely on rules to guide ethical behavior can be insufficient to address the moral dilemmas posed by complicated ethical situations that can emerge during research, at home or abroad.

### TCPS: the Canadian ethics environment

In Canada, ethical behaviour in human subjects research is guided by the Tri-Council Policy Statement (TCPS) on Ethical Conduct for Research Involving Humans. The TCPS is a 220-page document that aims to help readers “identify ethical issues in the design, conduct and oversight of research and to point the way to arriving at reasoned and ethical responses to these issues” ([3], p. 6). These guidelines are used by ethics boards to help assess proposed projects, however as a Canadian researcher, one must merely pass the TCPS 2: CORE (Course on Research Ethics) and receive ethics approval for their research project from their host University.

The TCPS 2: CORE is a 10-module online tutorial designed to familiarize researchers with the values, principles, and procedures all Canadian researchers are expected to follow. At the heart of the tutorial is an emphasis on minimizing the chances of physical, psychological, economic, and social harm and ensuring any such risks are proportionate to the perceived benefits, be they direct, indirect, or diffuse ([4], Module 3).

The tutorial describes the various constraints researchers are under when designing and executing their research. For example, researchers must ensure “all potential physical harms of a study have been identified and that measures are in place to minimize their occurrence and to offer follow-up care”, and they must be certain that “measures to eliminate or minimize [economic] risks are part of the study procedure, and that unavoidable risks are discussed with prospective participants in the consent process” ([4], Module 3).

Drawing on the TCPS, research ethics boards (REB) are tasked with ensuring researchers have thought through the implications of their study as it relates to various ethical values and that they have put in place procedures and constraints to ensure, for each study, that the path of least resistance for the researchers is ethical. To promote ethical outcomes, researchers must put in place rules bounding their research. In other words, the Canadian ethics environment is consequentialist and deontic in nature; to ensure ethical outcomes, it relies on the prescription of rules and obedience to those rules.

When Canadian researchers go into the field, it is only after they have gone through the process of designing their research to address particular issues identified by the REBs, and putting in place rules and constraints on their own research and behaviour to minimize the likelihood of behaving unethically given these issues. However, beyond the TCPS CORE training and receiving approval from the REB, researchers receive few resources to help them navigate complicated the ethical terrain that can arise. While the Belmont report recognizes that ethical issues are complex and rules come into conflict or are difficult to apply, after the REB approval process, there is little to no guidance about how to handle these situations.

I now turn to a discussion of a recent research project that demonstrates how easily such complicated situations can arise. I articulate the various ways in which a solely rules-based approach is insufficient in this case, and I argue for the importance of moving beyond a solely rule-based approach to ethical research involving human subjects.

## Methods

### Drinking and driving in South Africa

With over 13,000 road deaths in 2013, South Africa is one of the most dangerous places in the world to be on the road [5]. More concerning is the fact that an estimated 55–60% of these deaths involve alcohol. On paper, South Africa’s laws meet international standards; however, drinking and driving remains a huge problem in the country. The real issue, according to the World Health Organization (WHO), is one of “enforcement” [6].

In 2015, I submitted an ethics application to conduct research in South Africa on drinking and driving. The purpose of my research was to “investigate why South African drinkers continue to drink and drive in spite of the steep physical and legal risks associated with the practice.” The bulk of my research concerned understanding what South African drinkers (those actually at risk of drinking and driving) felt about the issue and their thoughts on why policies and interventions were not effective. To do so, I proposed an ethnographic methodology that included participant observation and short informal interviews: I proposed to go into bars and restaurants where people were drinking, observe the environment, and speak to drinkers about drinking and driving.

Two things were ethically problematic about my research from the start. The first was that I would be interviewing people who were potentially inebriated, or would become so, and thus could not properly consent to participating in the research. The second was that I would be in situations where illegal and dangerous activities might occur in a public and visible space—that is, people might end up drinking too much and going to drive home, putting me in an ethically problematic position.

It should be noted that the REB, in an attempt to save me from having to deal with these ethical issues, encouraged me to abandon the ethnographic approach in favour of an online survey. The REB made the case that it was unclear whether it was necessary to examine this particular population to answer my research questions. While a household survey or semi-structured interviews would have provided interesting data in a different context, there were several reasons why I advocated this approach here. The first was that I was interested in a specific population—drinkers who were likely to drive after drinking—and this was the best way to isolate this population. The second was that this approach allowed for snowball sampling and group discussion, allowing me to reach more individuals from the target population than did other methods. Finally, informal conversations in public spaces with a foreigner (who had a beer in his hand, no less) were less likely to raise suspicions that these efforts were coming from law enforcement. In short, I was confident that speaking to individuals in this context was the only way I would be able to get honest and frank discussions about drinking and driving from those most likely to engage in it. After some back-and-forth, it was decided that this methodology was acceptable and the application proceeded. Approval took over two months. It turned out, of course, that this research was destined to be as ethically challenging in practice as it was on paper—even with the rules and constraints put in place by the REB.

## Results

### Vignette #1: Jägerbombs and moral dilemmas

One evening, I was at a bar asking questions of a group of people. One of their friends arrived late and came to the table, curious about the strange foreigner. I explained who I was and what I was doing, and he said to me: “Sure! I’ll answer your questions, but first you’ll have to do a shot with us!” I declined and explained why that was not going to happen, but he insisted, and a few minutes later returned with a tray full of Jägerbombs^1^. I declined again, insisting it would be inappropriate for me to have the shot, and explaining that I would have to stop my questioning if people got drunk^2^.

The group downed their shots, and one remained. The purchaser then did his best to find someone else and pressured one of his friends to take the extra Jägerbomb. I had been speaking to the gentleman in question and knew he had driven to bar. I had seen him drink one beer and one shot, and now he was about to have another. This would put him well above the legal limit to drive in South Africa^3^.

I was thus in a position as a researcher where I was inadvertently contributing to a potential harm. As a participant in the space, I was expected to take the shot, and by not taking it, I created a situation where someone else would. Yet had I taken the shot, that would have put me over the limit myself, making it inappropriate to continue my research—I would have transitioned from researcher to bar patron and implicitly supported drinking and driving. Another option would have been to call out either the purchaser or the man about to drink the shot; however, this had the potential to cause social harm. Whatever path I chose, I was in complicated territory, facing an ethical dilemma that the REB did not provide clear guidance for.

This situation presented what I will call a *zungzwang* ethical dilemma. *Zungzwang* is a chess term for when a player must make a move, but every available move would leave the player worse off. For example, in the above scenario, every move has a consequence leading to an ethically problematic outcome, and there is no ability for the researcher to ‘pass’. Providing researchers with a list of rules to follow to ensure ethical behavior inadequately prepare researchers for *zungzwang* dilemmas because these dilemmas arise from interaction among the rules, not the rules themselves. That is, a *zungzwang* dilemma is the product of a situation in which following one rule necessitates breaking another, where any move is a bad move. These situations cannot always be determined a priori, and, as will be discussed below, it is unclear whether an overarching rule that would be compatible with the rules in question is even possible. In *zungzwang* ethical dilemmas, the burden of choice falls to the researcher. In other words, where the REB does not provide guidance, researchers must employ their own ethical framework to evaluate decisions concerning potential harms—and there is no guarantee that an individual’s ethical framework aligns with an REB’s.

### Vignette #2: drunk in love

Back to the scene that began this article. After witnessing an extremely drunk woman trying to persuade another to come home with her, I helped the first woman off the ground and suggested they take a taxi and retrieve the car tomorrow. I offered to call, and even pay for, the taxi.

The potential driver blew up at me, accused me of interfering with her attempts to bring this other woman home, and became angry with the other woman, who thought taking a taxi was a good idea. In essence, I had insulted this woman by questioning her ability to drive; I had embarrassed her in front of her crush and ruined her night. Far from defusing a potentially dangerous situation, my intervention (which looked great on paper…)^4^ resulted in this woman storming off, getting into her car, and speeding away, her tires squealing as she made a too-fast U-turn. So, my intervention, intended to prevent harm, resulted in a situation where this woman was driving not only drunk, but angry as well. My good intentions, while preventing the crush from getting into the car, also likely put the driver, and others, at higher risk of harm and even death.

I refer to this as a *contingent ethical dilemma*, where the ethical value of an action depends not on its content, but rather on its outcome. These situations are bound to come up in any research. Researchers are human and must make decisions during the course of their research, and these decisions—regardless of intent—can unwittingly lead to harm or contribute to situations where harm is more likely. Ultimately, there is no way of knowing whether one’s action contributes to or prevents harm, and there is nothing an ethics board can do to address this. Even if we can imagine potential moral dilemmas, to legislate all action beforehand is nonsensical, as the ethical value of the researcher’s action often depends not on the action itself, but rather on the result.

For example, consider a waiver for the use of someone’s photograph. The waiver has no ethical value in and of itself. An individual may not be able to read, may simply not read the waiver, or may misunderstand what it means. The waiver does not make the use of an individual’s photograph *ethical*, even though it may make it legal, or at least make an actor non-liable. What makes the waiver an ethical object is that it reflects consent. While the REB rule may be to obtain consent, the ethical indicator is the waiver, and it is entirely possible to have a waiver without consent or consent without a waiver. What matters here is not that the rules are followed, as indicated by the existence of a signed waiver, but that the consequence is ethical – that the individual understands and consents to what the waiver represents.

In the example of the drunk driver, my following of the rules potentially increased the individual’s risk, and these rules probably should not have been followed strictly in this case. Importantly, this experience also demonstrated that this rule could cause the exact harm it was trying to mitigate. This is extremely problematic when a researcher is provided only with a set of rules to follow, as it puts the researcher in a situation of being bound to follow harm-reduction rules while knowing they cause harm, and with no clarity on how to navigate this terrain.

It should be noted that my actions in the above two vignettes were completely ethical by the REB standards. Vignette #1 revealed a *zungzwang dilemma*, a situation in which different researchers could have behaved in completely different ways, with different outcomes, and yet would have remained compliant with REB guidelines. Vignette #2 showed a *contingent dilemma*, where the intervention was intended to reduce harm but did not. Contingent dilemmas may not be immediately apparent, but become clear after the fact and put researchers into situations where following the rules to reduce harm can actually increase it. These considerations, and others discussed below, give us reason to be skeptical about the value of a completely rule-based approach to research. As I will demonstrate using this case, the scope for a rule-based approach to research ethics is limited and must be supplemented in order to improve researchers’ ability to conduct ethical research.

## Discussion

### The limits of rule-based ethics

The above vignettes suggest a rule-based approach, while important, may be insufficient for resolving ethical dilemmas in research. I argue that it is indeed insufficient, for several reasons. First, and most broadly, it is not *desirable* to have an entirely rule-based approach to ethics. Second, even if it were desirable, it is not *possible* to completely articulate all the rules for a fully ethical, rule-based approach to research. Third, a rule-based approach is not necessarily *effective* at achieving the desired outcomes. Finally, ethical research requires different values that are not lexically ordered; thus application of the rules will not necessarily be *consistent*.

#### On the desirability of rule-based ethics

As Haggerty argues, the purpose of ethics boards—to formally manage the risks of conducting scientific inquiry—has given way to institutions that hamper critical research, encourage unethical behaviour, and homogenize and narrow our vision ([7], p. 412). Like any other bureaucratic organization, ethics boards have expanded in scope and size [8], with the result being that “the regulatory structure of the ethics bureaucracy is expanding outward, colonizing new groups, practices and institutions, while at the same time intensifying the regulation of practices deemed to fall within its official ambit” ([7], p. 394). In other words, more people are considered ‘researchers’, more activities are considered ‘research’, and ethics boards are involved in regulating more aspects of intellectual life.

One potential consequence is that ethics boards may begin to shape research to fit their own bureaucratic ends. For example, the REB suggested I pursue a survey-based approach rather than the ethically messy approach of talking to drinkers in bars. To their credit, I was able to convince them otherwise. However, it would have been much easier for the regulatory body (and for myself) had I simply proposed a survey. It also would have been easier had I proposed sending that survey to all drivers, not simply those who I had good reason to believe might be drinking. The potential for the tail to wag the dog here is quite high, given the power REBs have over the ability to conduct research.

Similarly, REBs may have different priorities than researchers and research populations when determining the rules. While the REB’s role towards the researcher is ostensibly to ensure the chances of harm among research participants are minimized, the REB does not exist in a vacuum; REBs also have a relationship with the universities they serve. Specifically, it is through REBs that universities can demonstrate their exercise of due diligence in ensuring researchers associated with the university will not engage in unethical behaviour. If a researcher does behave unethically, it will be in spite of the university’s best efforts to prevent this. In short, REBs have two objectives: to ensure ethical research occurs, and to limit the university’s liability in case it does not. They are not necessarily compatible, and there is a clear incentive to err on the side of caution [9].

Ultimately, we do everyone a disservice if research is ‘safe’ in terms of method and subject matter, and an insistence on a rule-based approach to ethical behaviour creates incentives to promote and conduct ‘safe’ research.

#### On the possibility of rule-based ethics

Even if a completely rule-based ethical approach to human subjects research is desirable, it is likely impossible, a point which the authors of the Belmont report recognized [1]. It is doubtful that all the rules an individual researcher must follow in the field can be determined a priori. This becomes even more difficult in contexts that the researcher (and the REB) is not particularly familiar with, such as research in foreign countries or research on underserved populations.

For example, it was highly unlikely that I would have anticipated being put into a situation like the one described in Vignette #1. Even if I had, should I have set rules concerning what I should do if the person insisted a non-driver take the shot? What if the shots had been light beers instead? The variations are infinite, and thus any rule-based approach to ethical behaviour implicitly relies on higher-order rules to guide behaviour.

One possibility would be to employ an overarching principle—a *categorical imperative* for human subjects research. The argument would be that there is one universal principle—say, do no harm—to which all other principles adhere. I find this position to be wholly unconvincing, given that there are competing objectives. They are not simply about being an ethical person; rather, they are about being an ethical researcher, and researchers must balance risks, harms, and rewards to different individuals, institutions, and research objectives. For example, it is acceptable to do some harm (e.g. deceive people) if the potential payoff is high. Indeed, the only imperatives I can imagine that would be suitable for research would not be about consequences (as the current ethics environment seems to promote), but rather about particular values—a subject to which we will return.

#### On the effectiveness of rule-based ethics

What is clear from both vignettes is that, as a researcher, it is possible to increase the probability of harm because one follows the rules explicitly. Thus, in the aforementioned situations and many others, it may be that the best thing a researcher can do to minimize risk to research participants is to *disobey* the ethics board’s rules. Indeed, once I know that following the rules will lead to unethical results, as an ethical individual, it becomes my obligation to disobey.

Consider Arendt’s portrait of Adolf Eichmann, the German bureaucrat tasked with running the trains on time during the Holocaust [10]. Arendt’s work clearly highlights the fact that actions cannot be divorced from consequences. Eichmann’s obedience made him a good bureaucrat, while also an accomplice to the murder of thousands. The fact that the consequences of his actions were unethical, even if the actions themselves were neutral or praised by the regime, should be enough to confirm Eichmann’s guilt. The logic should be the same for the researcher: if given the choice between following the rules or being ethical, a researcher should always choose being ethical. And if that is the case, then how should we understand the rules in place?

A solely rule-based approach to ethical research also does not allow for efforts at harm reduction. For example, if the rule says the researcher cannot allow someone who has been drinking to drive, what action should be taken when Person A has had eight beers and Person B has had two, and they insist they will not take a taxi? Obviously, the best option is that neither drives; however, as a person concerned with the welfare and well-being of my research subjects, I should do whatever I can to make sure the person who has had eight beers does not drive, even if it means encouraging the one who has had two beers (and is legally over the limit) to drive instead.

Any set of rules that allows the researcher to make subjective decisions about how to behave in particular situations is not exactly a useful rule-based framework. Subjectivity cannot be avoided; so, if we want our researchers to be ethical individuals, we *must* allow for subjective interpretation in the field, and that necessitates a different understanding of the application of rules to field research.

#### On the consistency of rule-based ethics

One possibility here is to codify and arrange the priorities of different spheres of value in relation to one another—to say, for instance, that *physical harm > psychological harm > social harm > economic harm* —and then provide consistent ways of measuring and assessing these things. This is of course, ridiculous. How many potential concussions in a population is greater than a possible loss of $10 each? How much possible embarrassment in front of your peers is worth $20? The assessment of (potential) risks and (potential) outcomes is completely subjective and relies on the judgments of both the researcher and the REB. The attempt to codify practice into rules by the REB is thus an attempt to codify a subjective decision into something to be consistently applied by the researcher.

One problem is that, while individual researchers might be consistent in their approach, any two researchers might not be consistent with each other. One REB might decide $20 is enough compensation for potential embarrassment, another might find that amount wholly inadequate. The relationship between potential risks and rewards, and how the likelihood of each is calculated, will be different in each case. Thus, two researchers could behave in different—even opposing—ways, and both would be seen as behaving ethically because they followed their ethics board’s rules.

There is also the issue of different norms or standards of lexical priority. Take, for example, a researcher who is taught to protect individuals against physical harm, social harm, and economic harm, in that order. Now imagine that person is conducting research in a community where the norms are different, where the individual’s physical well-being is less important than the well-being of the community, and where economic success is seen as a blessing from God and therefore a reflection of one’s moral worth and abilities to provide for one’s community. We can say then that this community’s priorities would be against social harm, economic harm, and physical harm, in that order.

In that setting, how is the researcher to behave if subjects adhere to these priorities? Say, they are willing to put themselves in harm’s way to participate in the research project because it comes with a large economic payoff? From the standpoint of those potential participants, they are acting in the most ethical way possible—putting their own safety at risk for the community’s benefit. For the researcher, this is unacceptably putting the participant in harm’s way for an economic benefit. When the researcher discovers the participant’s motivations (or that the research is physically risky for the participant) half-way through the research, which ethical standards apply and why?

#### Beyond ruled-based ethics

I argue above that a completely rule-based approach to ethics in human subjects research is not desirable, possible, or effective, and that any application of it is necessarily subjective and potentially inconsistent. However, the above discussion has also served as a reminder that underlying the rule-based approach of REBs is a set of particular values. Indeed, as per the Belmont report, this was always the purpose of REBs —to “identify the basic ethical principles that should underlie the conduct of biomedical and behavioral research involving human subjects and to develop guidelines which should be followed to assure that such research is conducted in accordance with those principles” ([1], p. 1). As we cannot hope to articulate all of the rules, and because we have competing spheres of values and actors (the individual, the research, the research subject, institutions, the community, etc.), it is not possible to articulate any *categorical imperative* for human subjects research. Indeed, even if we were able to correctly and consistently articulate the values to which researchers should subscribe, we would have immense difficulty in stating universally which ones to prioritize and when.

## Conclusions

### The virtuous researcher

The solution to manage complex situations, such as the one described above, is not to create more rules to constrain researchers, but rather to supplement these rules by creating the kind of researcher for which—when rules don’t work—rules are not necessary.

Imagine for a moment that the researcher is a toddler. This toddler wishes to play out back of the house, where there is a ravine. To minimize risk, the parents ask the child where she wishes to explore and then they determine what areas would be too dangerous. They put up a fence blocking the child from accessing the dangerous areas and then feel comfortable letting their child wander free in the backyard. This is roughly analogous to how REBs operate. Relative risks and rewards are discussed, and once approval has been granted, researchers are left to wander the field unmolested—provided they don’t try to climb the fence.

What if that fence is incomplete? Or poorly constructed? Or the land shifts beneath one of the poles? This is not going to happen in all situations, but could happen in some. Maybe the yard is too big, or the land too uneven, or it is in California in an area prone to earthquakes. There are many reasons not to trust the fence, no matter how large or sturdily constructed. When you have reason to believe the fence is not enough, what can you do?

The solution is either to not have children or to raise them so they are capable of behaving in such a way that a fence is not necessary. Instead of building a fence, ensure the children behave in accordance with the values that the fence represents. Then you can send them off into their backyard, or any backyard, without undue stress or worry.

What I am advocating here is the creation of virtuous researchers—virtuous in the Aristotelian sense. The virtuous researcher, like the virtuous individual, would be someone who “without relying on rules, is sensitive and intelligent enough to perceive what is noble or right as it varies from circumstance to circumstance” ([11], p. 178). Ethics boards and bodies should thus not only be concentrating on rules that constrain the individual from acting, but should also be developing within the individual “those inner traits, dispositions, and motives that qualify her as being virtuous” ([11], p. 177).

To create this ethical researcher is to abandon the focus on rules and constraints as the basis of ethical action. So, how do we create this virtuous researcher? Thomas Huw argues that a virtue ethics approach would “try to develop an idea of what kind of person a virtuous researcher is, rather than simply a listing of ‘dos and don’ts”’ ([12], p. 37). While “there is a place for rules of thumb to help people begin to appreciate the claims of morality …rules of thumb are just an early part of the process of moral development” ([12], p. 31). He argues that instead of rules and rules of thumb, “[s]tories, homilies, and examples are ways of getting people to see things in a certain way, a way of building an understanding which goes beyond the examples themselves and allows people to cope with new, perhaps unique, circumstances” ([12], p. 31).

Creating virtuous researchers will be a more effective way to address the problems outlined above with *zungzwang* and contingent ethical dilemmas. A researcher who is trained to *be* ethical will be better prepared to make the difficult decisions inherent in *zungzwang* dilemmas. Ethical researchers will also be better prepared to revise, adapt, and abandon rules when obedience to them will likely cause harm. When faced with a *contingent* ethical dilemma, an ethical person will always be preferred to someone who blindly follows the rules—and, while most researchers are not Eichmann, ethical behaviour is currently assessed by one’s compliance with rules and not by one’s alignment with ethical values.

As we cannot determine a priori whether a research environment will generate a moral dilemma, it is incumbent on us as ethical researchers to be prepared for moral dilemmas in general, not simply specific moral dilemmas that have been thought up ahead of time. To be clear, I am not arguing for abandoning rules in research ethics. Rules are useful, and in most cases, they will be sufficient. My concern here is with those situations where they are not—where rules come into conflict, or where it is discovered that following a rule has the opposite consequence than what it was designed to produce.

My argument is, rather, that in addition to articulating rules, ethics boards should do as much as possible to ensure researchers have clear guidelines when they go into the field. The rules they articulate will often be insufficient, and the way to address these limitations is to create virtuous rather than obedient researchers—that is, researchers who follow the spirit of the law, not just the letter. Truly ethical human subjects research would involve researchers who know the difference between right and wrong and do what is right, not researchers who simply do what the ethics board says is or is not ethical.

During this research, a respondent said to me: “You know, I am always fine to drive until that last drink.” Most things, including rules and guidelines for ethical behaviour, are useful and important until they are not. It is that moment when they are *not* useful that we should be concerned with, because, as I hope I have demonstrated, that moment likely occurs more often than we might want to admit. A focus on creating virtuous researchers, instead of more comprehensive ethical rules and guidelines, will do a tremendous amount to minimize the harm well-meaning researchers can cause.

How to create these virtuous individuals is a difficult question and one that will need to be addressed in future work. Possible strategies might be to focus more on mentorship and apprenticeship, or on habituation rather than simple training. For the moment, I am content to have made the case that human subjects research can present complicated ethical situations that REBs are not necessarily equipped to handle properly. Therefore, to be an ethical researcher requires thoughtful, moral, and virtuous researchers—not exceptionally competent bureaucrats.

## Abbreviations

CORE: Course on research ethics
REB: Research ethics boards TCPS: Tri-council policy statement
WHO: World Health Organization

## Footnotes

1 Jägerbombs, for the unfamiliar, are a shot of licorice-flavoured liquor dropped into an energy drink.

2 This was another concern of the REB, that once participants had granted consent they might become inebriated and no longer be in a position of sound mind to participate in the research. This concern turned out to be unfounded, however, as once an individual became too drunk to participate in the research, it was impossible to carry on a focused conversation with them. It was, in practice, simple to determine when a participant should no longer be questioned.

3 Though, depending on how long he stayed at the bar after that without drinking, he might have been fine to drive by the time he left.

4 The following is quoted verbatim from my approved application for ethics approval for this project: “There is one major potential concern that I see arising, and that is if a respondent appears to me to be intoxicated and reveals that they are planning on driving home. Ethically this is a difficult position to be in, as the respondent agreed to speak to me under the impression that they would not be put under any risk—including legal risk. Thus, I believe there would be a conflict if I were to call the police to report (potential) drunk driving. Instead, if this occurs, I will end the conversation immediately and insist that the individual take a taxi home. I will offer to telephone for a taxi for the individual, and if it is clear that the individual is only not taking a taxi due to financial constraints, I will pay for the taxi when it arrives. While participants will not receive compensation for participating in this research, I believe this is an acceptable solution that reduces the likelihood of physical harm, while also maintaining my commitment to not subjecting the respondent to legal or emotional harm. As the decision to drink and drive is that of the individual, and would not be encouraged by this conversation.”
